# Efficacy of ultrasound-guided microwave ablation combined with chemical ablation for uterine fibroid management

**DOI:** 10.3389/fonc.2025.1708837

**Published:** 2026-01-13

**Authors:** Yu Zhao, Tian-Pei Jiao, Yu-Xiao Zhang, Na Wang

**Affiliations:** 1 Department of Ultrasound, The First Hospital of Hebei Medical University, Shijiazhuang, Hebei, China; 2 Department of Smart Hospital Development, The First Hospital of Hebei Medical University, Shijiazhuang, Hebei, China

**Keywords:** chemical ablation, microwave ablation, recurrence, treatment efficacy, ultrasound guidance, uterine fibroids

## Abstract

**Background:**

Uterine fibroids are the most common benign tumors of the female reproductive tract and often cause menorrhagia, pelvic pain, and infertility. Ultrasound-guided microwave ablation (MWA) has gained recognition as a minimally invasive, uterus-preserving therapy; however, incomplete ablation may compromise long-term outcomes. This study aimed to evaluate the efficacy and safety of MWA combined with chemical ablation (CA) compared with MWA alone in patients with symptomatic fibroids.

**Methods:**

This retrospective cohort study included women aged 18–55 years with imaging-confirmed uterine fibroids treated between January 2021 and December 2024. Patients were allocated to either the MWA+CA group (n=126) or the MWA group (n=108). Both groups underwent ultrasound-guided percutaneous ablation procedures. Treatment efficacy was assessed based on fibroid volume reduction, cure rates, and recurrence. Statistical analyses included independent-samples t-tests for continuous variables and chi-square tests for categorical variables.

**Results:**

Both treatment groups showed significant reductions in fibroid volume. The MWA+CA group experienced a more pronounced reduction, with a mean volume decrease from 33.64 ± 8.20 cm³ to 10.37 ± 2.25 cm³ (p < 0.001), compared to the MWA group, where the volume decreased from 32.36 ± 7.18 cm³ to 15.36 ± 3.27 cm³ (p < 0.001). The overall effective rate in the MWA+CA group was 98.4%, significantly higher than the 88.9% in the MWA group (p = 0.002). Recurrence rates were significantly lower in the MWA+CA group (1.6% vs. 8.3%, p = 0.015).

**Conclusions:**

Ultrasound-guided MWA combined with CA is more effective in reducing fibroid volume and preventing recurrence compared to MWA alone. This combination offers a promising treatment option for symptomatic uterine fibroids, with no significant increase in adverse reactions. Larger, multicenter, prospective studies are needed to further validate these findings.

## 1 Introduction

Uterine fibroids, also referred to as leiomyomas, are the most common benign tumors of the female reproductive tract, with a lifetime prevalence exceeding 70% in women of reproductive age. Although nonmalignant, fibroids are associated with a spectrum of clinical manifestations, including menorrhagia, pelvic pressure, infertility, and recurrent pregnancy loss, which significantly impair quality of life (1, 2). Conventional management strategies encompass pharmacologic therapies, minimally invasive interventions, and surgical procedures such as hysterectomy and myomectomy. Despite the availability of these modalities, optimal treatment remains challenging due to limitations in efficacy, recurrence risk, procedure-related morbidity, and the need for fertility preservation in younger patients (3–5).

Over the past two decades, minimally invasive thermal ablation technologies, including radiofrequency ablation, high-intensity focused ultrasound (HIFU), and microwave ablation (MWA), have gained increasing attention as alternatives to conventional surgery. Among these, MWA is characterized by rapid heating, high thermal efficiency, and larger ablation volumes, thereby enabling effective coagulative necrosis of targeted fibroid tissue with reduced procedure time compared to other energy-based modalities. Ultrasound guidance further enhances the precision of MWA, allowing real-time visualization of fibroid margins, monitoring of ablation zones, and minimizing damage to surrounding normal myometrium (6, 7). Nevertheless, residual viable tissue at the periphery of the ablation zone remains a major cause of incomplete treatment and subsequent recurrence. To address this limitation, combined therapeutic strategies have been proposed. Chemical ablation (CA), achieved through the intralesional injection of sclerosing or cytotoxic agents such as absolute ethanol or lauromacrogol, induces ischemic necrosis and fibrotic remodeling of fibroid tissue. When integrated with thermal ablation, chemical agents can extend the ablation boundary, enhance tissue devascularization, and reduce the likelihood of viable residual nodules. Previous studies in hepatic tumors and thyroid nodules have demonstrated that the synergistic use of thermal and CA improves ablation completeness and reduces recurrence rates (8–10). However, limited evidence is available regarding the combined application of ultrasound-guided MWA and CA in the management of uterine fibroids.

The aim of this study was to evaluate the clinical efficacy and safety of ultrasound-guided MWA combined with CA for the treatment of symptomatic uterine fibroids. Specifically, we sought to compare the outcomes of MWA+CA versus MWA alone in terms of fibroid volume reduction, treatment efficacy, recurrence rates, and adverse events. Additionally, we aimed to assess the potential benefits of combining CA with microwave treatment, as well as to explore the impact of fibroid characteristics such as location, size, and vascularity on treatment outcomes. This study contributes to the growing body of evidence supporting minimally invasive, fertility-preserving interventions for the management of uterine fibroids.

## 2 Methods

### 2.1 Study design

This retrospective cohort study included patients who underwent treatment for uterine fibroids at our institution between January 2021 and December 2024. Eligible participants were women aged 18 to 55 years with a definitive diagnosis of uterine fibroids confirmed by imaging modalities, including transvaginal or transabdominal ultrasonography and/or magnetic resonance imaging. Only patients with fibroids measuring 2–10 cm in maximum diameter that were technically suitable for percutaneous ablation under ultrasound guidance. Patients were excluded if they presented with acute pelvic or systemic infection, severe coagulopathy or concurrent anticoagulant therapy that could not be safely discontinued, significant cardiopulmonary or hepatic dysfunction precluding intervention, or a documented allergy or intolerance to the chemical agents used in ablation. According to the treatment protocol, enrolled patients were allocated into two groups: those who received ultrasound-guided MWA combined with CA (MWA+CA group) and those who underwent MWA alone (MWA group). Informed consent was obtained from all participants. The study protocol was reviewed and approved by the hospital’s ethics committee. All procedures were conducted in accordance with relevant guidelines and the Declaration of Helsinki. All data were anonymized prior to analysis to ensure participant confidentiality.

### 2.2 Intervention protocols

MWA+CA Group: Patients in the combined treatment group underwent ultrasound-guided MWA followed by CA. All procedures were performed using the Siemens Acuson S2000 color Doppler ultrasound diagnostic system (Siemens, Germany) equipped with a 2.5–4.5 MHz abdominal probe and a 4–9 MHz transvaginal probe for real-time image guidance. The ablation system consisted of the KY-2000 series MWA device (Nanjing Kangyou Medical Technology Co., China), operating at a frequency of 2450 MHz, with 14G MWA needles of 15, 20, or 25 cm in length. Prior to treatment, ultrasound scanning was performed to determine the puncture site, assess fibroid size, location, and adjacent structures, and evaluate vascularity with contrast-enhanced ultrasound. For patients with unfavorable abdominal conditions, a transvaginal route was selected. All procedures were performed under intravenous basal anesthesia with local infiltration of 0.1% lidocaine at the puncture site. After routine skin or perineal disinfection and sterile draping, puncture was carefully carried out under real-time ultrasound guidance, avoiding major vessels, the bladder, intestines, and the endometrium. The microwave needle was inserted into the lesion, and ablation was performed from the deep to superficial layers and from the center to the periphery. The microwave output power was set at 45–55 W. Real-time ultrasound monitoring was used to observe echogenic changes within the ablation zone. When gas-like hyperechoic changes extended to approximately 0.5–1.0 cm beyond the planned ablation margin, energy delivery was terminated. If bowel loops approached the treatment area or if visualization was compromised, ablation was immediately suspended. Following completion of MWA, areas adjacent to critical structures that were not adequately ablated were targeted for CA. A 19G PTC cannula needle (Hakko, Japan) was inserted subcapsularly into the residual fibroid margin under ultrasound guidance. Lauromacrogol injection (polidocanol, Tianyu Pharmaceutical Co., China; 10 mL per ampoule, 100 mg) was administered at a volume of 5–10 mL. The endpoint of injection was the appearance of a diffuse, cloud-like hyperechoic pattern extending around the fibroid capsule, indicating adequate agent distribution. All patients were monitored postoperatively for vital signs and immediate complications.

Following completion of MWA, a waiting period of approximately 3–5 minutes was routinely applied to allow partial dissipation of gas-induced hyperechoic artifacts. During this interval, the ultrasound probe was dynamically repositioned to optimize the acoustic window. Before advancing the CA needle, the operative field was reassessed in real time to identify residual non-ablated margins, particularly subcapsular viable tissue and areas adjacent to bowel, bladder, or major vessels. These peripheral regions typically exhibited relatively hypoechoic characteristics compared with the central ablation core, enabling reproducible identification despite partial artifact persistence. The fibroid capsule, which remained clearly visualized even in the presence of intralesional gas, served as the primary anatomical landmark to guide safe subcapsular insertion of the 19G PTC cannula needle (Hakko, Japan). When necessary, fan-shaped sweeping of the probe, variation of insonation angle, and switching between abdominal and transvaginal approaches were employed to bypass localized gas shadows and restore visualization. A 19G PTC cannula needle (Hakko, Japan) was inserted subcapsularly into the residual fibroid margin under ultrasound guidance. Lauromacrogol injection (polidocanol, Tianyu Pharmaceutical Co., China; 10 mL per ampoule, 100 mg) was administered at a volume of 5–10 mL. Adequate CA was confirmed by observing a diffuse, cloud-like hyperechoic distribution spreading along the fibroid capsule during injection, serving as a secondary safety checkpoint to verify proper needle placement and effective agent delivery. All patients were monitored postoperatively for vital signs and immediate complications.

MWA Group: Patients in the monotherapy group underwent ultrasound-guided MWA following the same imaging system and procedural preparation as described above. Under intravenous basal anesthesia and local infiltration with 0.1% lidocaine, percutaneous or transvaginal puncture was performed under real-time ultrasound guidance. The ablation needle (14G, 15–25 cm) was advanced into the fibroid, and ablation was conducted sequentially from the deep to superficial regions and from the center outward, with microwave power set at 45–55 W. Real-time ultrasound was employed to monitor the ablation zone, and energy delivery was terminated once the hyperechoic changes reached 0.5–1.0 cm beyond the preplanned boundary. Care was taken to avoid injury to adjacent viscera, major vessels, and the endometrium. No chemical agent was administered in this group. Post-procedural monitoring of vital signs and immediate complications was performed in the same manner as in the combined group.

### 2.3 Observation indicators

Treatment efficacy was assessed based on a clinical response classification system commonly used in uterine fibroid ablation studies, adapted to fit the context of our study (11). Patients were classified into three categories based on the percentage reduction in fibroid volume, as measured by ultrasonography, and clinical symptom improvement: Cure (Markedly Effective): Patients in this group showed complete symptom relief with fibroid volume reduction ≥60% on ultrasound examination; Effective: Patients who exhibited clinical symptom improvement with fibroid volume reduction between 30% and 59%; Ineffective: Patients whose fibroid volume reduction was <30% or who did not show any clinical symptom improvement. The total effective rate was calculated by summing the cure and effective cases.

Fibroid volume was measured using two-dimensional ultrasonography. For each lesion, the maximum longitudinal diameter (D1), anteroposterior diameter (D2), and transverse diameter (D3) were obtained in orthogonal planes. Volume was calculated using the standard ellipsoid formula: Volume=D1×D2×D3×0.523. All measurements were performed by experienced sonographers, and each parameter was recorded three times with the mean value used for analysis to minimize intraobserver variability.

### 2.4 Follow-up evaluation

Follow-up information was obtained retrospectively through the hospital electronic medical record system. For each patient, all available outpatient visits, imaging examinations, and telephone follow-up records were reviewed. Although the institutional protocol recommends ultrasonography at 1, 3, 6, and 12 months after treatment and annually thereafter, the actual follow-up duration for analysis was determined by the most recent documented clinical or imaging encounter available in the electronic database. Recurrence was defined as either: (1) ultrasonographic evidence of viable regrowth within the treated lesion, or (2) the development of new symptomatic fibroids requiring further management. For recurrence analysis, the follow-up period for each patient was calculated from the date of the index ablation procedure to the date of the last recorded ultrasound or clinical assessment. This allowed accurate determination of individual follow-up durations, regardless of variations in actual visit intervals.

### 2.5 Statistical analysis

All statistical analyses were performed using SPSS software (version 28.0; IBM Corp., Armonk, NY, USA). Continuous variables, such as fibroid volume, were expressed as mean ± standard deviation (SD). Normality of distribution was assessed using the Shapiro–Wilk test. For normally distributed data, comparisons between the two groups were conducted using the independent-samples t-test, while within-group pre- and post-treatment comparisons were evaluated using the paired-samples t-test. Non-normally distributed data were analyzed using the Mann–Whitney U test for intergroup comparisons and the Wilcoxon signed-rank test for intragroup comparisons. Categorical variables, including treatment efficacy (markedly effective, effective, ineffective), overall response rate, and incidence of adverse reactions, were expressed as frequencies and percentages. Comparisons between groups were performed using the chi-square test or Fisher’s exact test, as appropriate. Subgroup comparisons were performed using one-way Analysis of Variance (ANOVA) or Kruskal-Wallis test for continuous variables, depending on normality, and chi-square or Fisher’s exact test for categorical variables. A two-sided p-value < 0.05 was considered to indicate statistical significance.

## 3 Results

### 3.1 Baseline characteristics of patients

A total of 234 patients with symptomatic uterine fibroids were included in the analysis, comprising 126 in the MWA+CA group and 108 in the MWA group. Baseline demographic and clinical characteristics are summarized in **Table 1**. The mean age was comparable between the two groups (41.2 ± 6.5 years vs. 40.8 ± 6.7 years, p = 0.644). Similarly, there was no significant difference in body mass index (23.7 ± 2.8 kg/m² vs. 23.4 ± 2.6 kg/m², p = 0.399). The distribution of parity did not differ significantly between groups, with the majority of patients being parous (69.8% in the MWA+CA group vs. 68.5% in the MWA group, p = 0.827). With respect to fibroid-related parameters, the mean maximum fibroid diameter was 5.3 ± 1.4 cm in the MWA+CA group and 5.2 ± 1.5 cm in the MWA group, showing no statistical difference (p = 0.599). The mean disease duration was also similar between groups (3.8 ± 2.1 years vs. 3.6 ± 2.3 years, p = 0.488). In addition, baseline hemoglobin levels were comparable (108.6 ± 12.3 g/L vs. 107.4 ± 13.1 g/L, p = 0.471). Overall, no statistically significant differences were observed between the MWA+CA and MWA groups across all baseline demographic and clinical variables.

**Table 1:** Baseline characteristics of patients in the MWA+CA group and MWA group.

Variable	MWA+CA group (n = 126)	MWA group (n = 108)	Test statistic	p-value
Age (years), mean ± SD	41.2 ± 6.5	40.8 ± 6.7	t = 0.462	0.644
Body mass index (kg/m²), mean ± SD	23.7 ± 2.8	23.4 ± 2.6	t = 0.844	0.399
Parity (n, %)			χ² = 0.048	0.827
0	38 (30.2)	34 (31.5)		
≥1	88 (69.8)	74 (68.5)		
Maximum fibroid diameter (cm), mean ± SD	5.3 ± 1.4	5.2 ± 1.5	t = 0.527	0.599
Baseline hemoglobin (g/L), mean ± SD	108.6 ± 12.3	107.4 ± 13.1	t = 0.722	0.471
Disease duration (years), mean ± SD	3.8 ± 2.1	3.6 ± 2.3	t = 0.695	0.488

MWA, Microwave ablation; CA, Chemical ablation; SD, Standard Deviation; BMI, Body Mass Index.

### 3.2 Effect of MWA+CA versus MWA on fibroid volume reduction

As shown in **Table 2**, both groups demonstrated a significant reduction in fibroid volume following treatment. In the MWA+CA group, the mean fibroid volume decreased markedly from 33.64 ± 8.20 cm³ before treatment to 10.37 ± 2.25 cm³ after treatment, representing a substantial reduction (t = 30.721, p < 0.001). Similarly, in the MWA group, fibroid volume was reduced from 32.36 ± 7.18 cm³ at baseline to 15.36 ± 3.27 cm³ after treatment, and this decrease was also statistically significant (t = 22.393, p < 0.001). When comparing the two groups, baseline fibroid volumes were not statistically different (t = 1.260, p = 0.209), indicating good comparability prior to intervention. However, post-treatment fibroid volumes were significantly smaller in the MWA+CA group compared with the MWA group (t = 13.753, p < 0.001). These findings suggest that while both treatments are effective in reducing fibroid size, the addition of CA to MWA provides a more pronounced therapeutic benefit.

**Table 2:** Changes in uterine fibroid volume before and after treatment in the two groups (cm³).

Group	n	Pre-treatment (mean ± SD)	Post-treatment (mean ± SD)	t value	p value
MWA+CA group	126	33.64 ± 8.20	10.37 ± 2.25	30.721	<0.001
MWA group	108	32.36 ± 7.18	15.36 ± 3.27	22.393	<0.001
t (between groups)		1.260	13.753		
p (between groups)		0.209	<0.001		

SD, Standard deviation; MWA, Microwave ablation; CA, Chemical ablation.

### 3.3 Treatment efficacy and recurrence

As presented in **Table 3**, both groups achieved favorable therapeutic outcomes following intervention, but the efficacy was more pronounced in the MWA+CA group. In this group (n = 126), 106 patients (84.1%) were classified as cured and 18 (14.3%) as effective, with only 2 patients (1.6%) considered ineffective. This corresponded to an overall effective rate of 98.4%. In comparison, the MWA group (n = 108) showed 76 patients (70.4%) classified as cured, 20 (18.5%) as effective, and 12 (11.1%) as ineffective, resulting in an overall effective rate of 88.9%. Between-group analysis revealed statistically significant differences in cure rate (χ² = 6.367, p = 0.012), ineffective rate (χ² = 9.377, p = 0.002), and overall effective rate (χ² = 9.377, p = 0.002), all favoring the MWA+CA group. The difference in the proportion of patients categorized as “effective” did not reach statistical significance (p = 0.382). With respect to recurrence, the MWA+CA group demonstrated a markedly lower recurrence rate of 1.6% compared with 8.3% in the MWA group (χ² = 5.908, p = 0.015). This finding further supports the superiority of combined treatment in achieving durable therapeutic effects.

**Table 3:** Treatment efficacy and recurrence in the two groups.

Group	n	Cure n (%)	Effective n (%)	Ineffective n (%)	Overall effective n (%)	Recurrence n (%)
MWA+CA group	126	106 (84.1)	18 (14.3)	2 (1.6)	124 (98.4)	2 (1.6)
MWA group	108	76 (70.4)	20 (18.5)	12 (11.1)	96 (88.9)	9 (8.3)
χ² (between groups)		6.367	0.766	9.377	9.377	5.908
p (between groups)		0.012	0.382	0.002	0.002	0.015

MWA, Microwave ablation; CA, Chemical ablation.

### 3.4 Adverse reactions

The incidence of treatment-related adverse reactions in both groups is summarized in **Table 4**. Overall, the frequency of complications was low and comparable between the two groups, with no statistically significant differences observed. In the MWA+CA group (n = 126), nausea and vomiting occurred in 20 patients (15.9%), abdominal pain or diarrhea in 21 patients (16.7%), vaginal discharge in 6 patients (4.8%), and fever in 12 patients (9.5%). Similarly, in the MWA group (n = 108), the rates of nausea and vomiting, abdominal pain or diarrhea, vaginal discharge, and fever were 16.7%, 15.7%, 4.6%, and 10.2%, respectively. Statistical comparisons revealed no significant differences between the two groups across all adverse events, including nausea/vomiting (χ² = 0.027, p = 0.870), abdominal pain/diarrhea (χ² = 0.037, p = 0.848), vaginal discharge (χ² = 0.002, p = 0.962), and fever (χ² = 0.029, p = 0.866).

**Table 4:** Adverse reactions in the two groups.

Group	n	Nausea/vomiting n (%)	Abdominal pain/diarrhea n (%)	Vaginal discharge n (%)	Fever n (%)
MWA+CA group	126	20 (15.9)	21 (16.7)	6 (4.8)	12 (9.5)
MWA group	108	18 (16.7)	17 (15.7)	5 (4.6)	11 (10.2)
χ² (between groups)		0.027	0.037	0.002	0.029
p (between groups)		0.870	0.848	0.962	0.866

MWA, Microwave ablation; CA, Chemical ablation.

### 3.5 Subgroup analysis of fibroid location, distance to endometrium, and vascularity

Subgroup analysis based on fibroid location showed that fibroids located in the posterior uterus had a significantly greater volume reduction in the MWA+CA group compared with the MWA group (71.5% vs. 52.7%, p = 0.039). For anterior uterine fibroids, the volume reduction was 70.3% in the MWA+CA group and 51.3% in the MWA group (p = 0.063). Fundal fibroids showed reductions of 67.8% and 49.1% in the MWA+CA and MWA groups, respectively (p = 0.121). In the subgroup stratified by distance to the endometrium, fibroids located <2 cm from the endometrium exhibited a reduction of 70.8% in the MWA+CA group, compared with 51.5% in the MWA group (p = 0.027). For fibroids located 2–4 cm from the endometrium, the reductions were 69.3% and 52.0% in the two groups, respectively (p = 0.071). When the distance exceeded 4 cm, the reductions were 65.9% in the MWA+CA group and 47.9% in the MWA group (p = 0.103). Analysis based on vascularity indices showed that fibroids with high vascularity had a volume reduction of 72.8% in the MWA+CA group compared with 53.0% in the MWA group (p < 0.001). For fibroids with moderate vascularity, volume reductions were 69.2% and 52.1% in the respective groups (p = 0.014) (**Supplementary Tables 1**-**3**).

### 3.6 Follow-up duration

All 234 patients had available follow-up records in the institutional database. Based on the time from treatment to the last documented clinical or imaging evaluation, the median follow-up duration was 23 months (range: 12–38 months) in the MWA+CA group and 22 months (range: 12–36 months) in the MWA group (p > 0.05).

## 4 Discussion

This retrospective cohort study evaluated ultrasound-guided MWA+CA versus MWA alone for symptomatic uterine fibroids. Baseline demographic and clinical variables were balanced between groups, minimizing confounding. Three principal findings emerged. First, both strategies achieved significant post-procedural volume reduction, with a greater absolute decrease in the MWA+CA group. Second, clinical response favored combination therapy, reflected by higher cure and overall effective rates and a lower proportion of ineffective cases. Third, the recurrence rate was significantly lower after MWA+CA, while the safety profile, assessed by common post-procedural adverse reactions, was comparable between groups. Collectively, these data indicate that adjunctive CA augments the therapeutic effect of MWA without compromising tolerability.

The superiority of MWA+CA is biologically plausible. MWA generates uniform dielectric heating, producing rapid coagulative necrosis, but residual viable tissue may persist at the periphery due to heat-sink effects and heterogeneous perfusion. Intralesional sclerosants such as lauromacrogol (polidocanol) can diffuse into microvascular and interstitial compartments at the treatment boundary, causing endothelial injury, microthrombosis, and ischemic necrosis. When deployed immediately after thermal ablation, a chemical agent may (i) extend the effective margin beyond thermal isotherms, (ii) reduce perfusion-mediated heat loss in partially ablated tissue, and (iii) remodel the microvasculature to limit post-treatment reperfusion. This complementary mechanism provides a coherent explanation for the greater post-treatment volume reduction, higher cure rate, and lower recurrence observed in the MWA+CA group. The absence of excess adverse reactions in MWA+CA suggests that, when administered subcapsularly under real-time ultrasound guidance with attention to endometrial proximity, the incremental risk of sclerosant injection is acceptable (12–14). However, it should be noted that the recurrence analysis was based on a median follow-up of 23 months. Given the benign and slow-growing nature of uterine fibroids, this duration may not fully capture long-term recurrence dynamics. Therefore, the observed lower recurrence rate in the MWA+CA group should be interpreted with caution and confirmed through studies with extended follow-up periods.

Recent prospective and retrospective series have consolidated the role of ultrasound-guided MWA as a uterus-sparing treatment for fibroids. A single-center cohort (2018–2022) reported clinically meaningful reductions in fibroid volume and symptom burden at 12 months after percutaneous MWA, with parallel improvements in hemoglobin and health-related quality of life (13). A more recent prospective study from a Scandinavian center (2020–2023) found significant symptom relief on UFS-QoL at six months and high acceptability following percutaneous or transvaginal ultrasound-guided MWA, supporting real-world feasibility (15). Emerging technique papers also describe planning aids (e.g., three-dimensional visualization platforms) to optimize needle trajectory and ablation geometry for large myomas, underscoring the modality’s continued refinement (16). Case-based evidence similarly documents symptom control with targeted devascularization of feeding vessels by MWA (17). Direct evidence on combining thermal and CA in uterine fibroids remains limited but conceptually consistent with data from adjacent fields. In a clinical protocol evaluating adjuncts to HIFU, pre-procedural intratumoral ethanol was used to create a necrotic core before sonication; although the trial’s primary comparison centered on oxytocin add-on, it illustrates a pragmatic framework for chemical-thermal sequencing in leiomyomas (18). More broadly, oncology literature recognizes synergy when chemical or endovascular devascularization is paired with thermal ablation, improving local control by mitigating heat-sink effects and enhancing margin lethality (19). Within gynecology, contemporary reviews emphasize the expanding portfolio of minimally invasive, image-guided treatments and the need for individualized selection based on fibroid burden and reproductive goals, a context in which an efficacious, uterus-preserving combination strategy has practical appeal (20). The present study adds to this literature by providing comparative clinical data that the addition of CA to MWA not only augments short-term response (volume reduction, cure and overall effective rates) but also confers a lower early recurrence rate without increasing adverse events. These observations align with the theoretical advantages of combination therapy and extend prior single-modality MWA reports by demonstrating an incremental benefit on durability.

Lauromacrogol (polidocanol) is a non-ionic surfactant with sclerosing, anesthetic, and endothelial-disruptive properties, traditionally used for intravascular sclerotherapy. Recent preclinical and limited clinical studies suggest its potential efficacy for interstitial use in solid tissues, including thyroid nodules, benign breast lesions, and hepatic cysts (21). Its mechanism involves both direct cytotoxicity through cell membrane disruption and ischemic necrosis induced by microvascular occlusion (22). This supports its use as an adjunct in fibroid ablation by enhancing tissue coagulation and preventing recurrence. While the interstitial application of lauromacrogol in this study is off-label, prior studies confirm its local cytotoxicity and fibrotic remodeling effects when injected under ultrasound guidance, with no significant systemic toxicity at low doses (23). Given the limited large-scale data for uterine fibroid ablation, this study contributes novel insights into its safety and potential efficacy. The primary endpoint of this study, fibroid volume reduction, was objectively assessed using two-dimensional ultrasonography, offering a standardized and reliable metric for treatment efficacy. Fibroid volume reduction correlates directly with the size of the fibroid and symptom improvement, making it a crucial indicator of therapeutic success. Both treatment groups demonstrated significant volume reductions, with the MWA+CA group showing a more pronounced decrease compared to MWA alone. These findings highlight the added benefit of combining CA with microwave treatment. While clinical symptom improvement is important, radiological measures like volume reduction provide a more objective and quantifiable assessment of therapeutic impact, which is especially critical in minimally invasive procedures like ablation, where precise monitoring of tissue changes is necessary for long-term treatment evaluation.

For women seeking uterus-preserving management of symptomatic fibroids, ultrasound-guided MWA+CA represents a rational escalation from thermal monotherapy. The combination yields greater debulking and higher overall response, with a reduction in early recurrence and no detected increase in common adverse reactions. Clinically, MWA+CA may be prioritized for larger, hypervascular, or recurrent nodules in which peripheral viability after thermal ablation alone is a concern, particularly when rapid recovery and preservation of reproductive potential are goals. Integration into multidisciplinary pathways can broaden minimally invasive options while maintaining safety parameters consistent with outpatient or short-stay care (24, 25). Strengths include (i) a relatively large, contemporaneous cohort for a single-center image-guided series; (ii) balanced baseline characteristics that reduce measured confounding; (iii) standardized ultrasound-guided techniques with clearly defined ablation parameters; and (iv) comprehensive outcomes encompassing objective volume change, categorical clinical response, recurrence, and adverse events. The study thus provides an internally consistent comparison of MWA+CA versus MWA.

Limitations merit consideration. While baseline demographics were comparable between the two treatment groups, potential unmeasured confounders, such as fibroid vascularity on contrast-enhanced ultrasound, FIGO classification, and baseline symptom severity, may have influenced outcomes. These factors should be considered when interpreting the results. Future prospective studies with more comprehensive data collection are needed to address these confounders and assess their impact on treatment outcomes. The retrospective nature of this study limited our ability to perform further stratification by FIGO classification, fibroid location, or vascularity indices, which may interact with chemical-thermal synergy. Although subgroup analyses provided valuable insights, a prospective, standardized protocol is required to validate these findings and refine our understanding of fibroid subtypes’ influence on treatment efficacy. Another limitation is the inability to evaluate the relationship between fibroid internal composition and treatment response, particularly regarding tissue characteristics. Future research incorporating advanced imaging techniques, such as quantitative MRI or ultrasound radiomics, is needed to explore how fibroid composition affects ablation outcomes. Although the risk of occult malignancy in our cohort was extremely low due to the benign nature of the fibroids and exclusion of high-risk imaging profiles, this risk cannot be fully excluded in a retrospective study. Future studies should include more rigorous screening for malignancy to ensure patient safety. Finally, the single-center design and limited follow-up period may affect the generalizability of the findings. While recurrence was reduced in the MWA+CA group, the follow-up duration may not capture late regrowth or the need for reintervention beyond the study window. Larger, multicenter, prospective studies with longer follow-up and pre-specified reproductive outcomes are needed to validate the durability and generalizability of these results.

## 5 Conclusions

Ultrasound-guided MWA combined with CA achieved greater fibroid volume reduction, higher cure and overall effective rates, and lower recurrence than MWA alone, with a comparable adverse-event profile. These findings support MWA+CA as an efficacious, uterus-preserving option for symptomatic leiomyomas. Prospective, multicenter studies with standardized protocols and longer follow-up are warranted to validate durability and refine patient selection.

## Data Availability

The raw data supporting the conclusions of this article will be made available by the authors, without undue reservation.
