# Supplementary material for: Efficacy of ultrasound-guided microwave ablation combined with chemical ablation for uterine fibroid management
Source: Front Oncol. 2026 Jan 13;15:1708837. doi: 10.3389/fonc.2025.1708837 (PMC12834760; doi:10.3389/fonc.2025.1708837)
Supplement: Supplementary file 1 [file Table1.docx]

Supplementary Table 1. Subgroup Analysis of Fibroid Location and Treatment Efficacy

| Fibroid Location | Group | n | Pre-treatment Volume (mean ± SD) | Post-treatment Volume (mean ± SD) | Volume Reduction (%) | p-value |
| --- | --- | --- | --- | --- | --- | --- |
| Posterior Uterus | MWA+CA | 45 | 33.80 ± 8.40 | 9.60 ± 2.20 | 71.50% | 0.039 |
|  | MWA | 40 | 32.50 ± 7.80 | 15.40 ± 3.10 | 52.70% |  |
| Anterior Uterus | MWA+CA | 42 | 34.00 ± 8.30 | 10.10 ± 2.30 | 70.30% | 0.063 |
|  | MWA | 38 | 32.00 ± 7.10 | 15.60 ± 3.20 | 51.30% |  |
| Fundal Uterus | MWA+CA | 39 | 33.50 ± 8.10 | 10.80 ± 2.40 | 67.80% | 0.121 |
|  | MWA | 30 | 31.90 ± 7.50 | 16.20 ± 3.10 | 49.10% |  |

Abbreviations:

MWA+CA: Microwave Ablation Combined with Chemical Ablation

MWA: Microwave Ablation

SD: Standard Deviation

Supplementary Table 2. Subgroup Analysis Based on Distance to Endometrium and Treatment Efficacy

| Distance to Endometrium | Group | n | Pre-treatment Volume (mean ± SD) | Post-treatment Volume (mean ± SD) | Volume Reduction (%) | p-value |
| --- | --- | --- | --- | --- | --- | --- |
| <2 cm | MWA+CA | 56 | 33.60 ± 8.10 | 9.80 ± 2.30 | 70.80% | 0.027 |
|  | MWA | 52 | 32.40 ± 7.60 | 15.70 ± 3.20 | 51.50% |  |
| 2–4 cm | MWA+CA | 45 | 34.10 ± 8.30 | 10.50 ± 2.20 | 69.30% | 0.071 |
|  | MWA | 46 | 33.20 ± 7.90 | 16.00 ± 3.00 | 52.00% |  |
| >4 cm | MWA+CA | 16 | 32.90 ± 7.80 | 11.20 ± 2.40 | 65.90% | 0.103 |
|  | MWA | 19 | 31.70 ± 7.40 | 16.50 ± 3.10 | 47.90% |  |

Abbreviations:

MWA+CA: Microwave Ablation Combined with Chemical Ablation

MWA: Microwave Ablation

SD: Standard Deviation

Supplementary Table 3. Subgroup Analysis Based on Vascularity Indices and Treatment Efficacy

| Vascularity Indices | Group | n | Pre-treatment Volume (mean ± SD) | Post-treatment Volume (mean ± SD) | Volume Reduction (%) | p-value |
| --- | --- | --- | --- | --- | --- | --- |
| High Vascularity | MWA+CA | 70 | 33.90 ± 8.20 | 9.20 ± 2.10 | 72.80% | <0.001 |
|  | MWA | 62 | 32.50 ± 7.50 | 15.30 ± 3.10 | 53.00% |  |
| Moderate Vascularity | MWA+CA | 50 | 34.30 ± 8.40 | 10.60 ± 2.20 | 69.20% | 0.014 |
|  | MWA | 52 | 33.80 ± 7.80 | 16.20 ± 3.30 | 52.10% |  |

Abbreviations:

MWA+CA: Microwave Ablation Combined with Chemical Ablation

MWA: Microwave Ablation

SD: Standard Deviation
